# Biomarker significance of plasma and tumor miR-21, miR-221, and miR-106a in osteosarcoma

**DOI:** 10.18632/oncotarget.18236

**Published:** 2017-05-27

**Authors:** Manjula Nakka, Wendy Allen-Rhoades, Yiting Li, Aaron J. Kelly, Jianhe Shen, Aaron M. Taylor, Donald A. Barkauskas, Jason T. Yustein, Irene L. Andrulis, Jay S. Wunder, Richard Gorlick, Paul S. Meltzer, Ching C. Lau, Tsz-Kwong Man, the TARGET osteosarcoma consortium

**Affiliations:** ^1^ Texas Children’s Cancer and Hematology Centers, Texas Children’s Hospital, Houston, TX, USA; ^2^ Department of Pediatrics, and Baylor College of Medicine, Houston, TX, USA; ^3^ Program of Structural and Computational Biology and Molecular Biophysics, Baylor College of Medicine, Houston, TX, USA; ^4^ Dan L. Duncan Cancer Center, Baylor College of Medicine, Houston, TX, USA; ^5^ Department of Preventive Medicine, Keck School of Medicine, University of Southern California, Los Angeles, CA, USA; ^6^ Children’s Oncology Group, Monrovia, CA, USA; ^7^ Lunenfeld-Tanenbaum Research Institute, Sinai Health System, Toronto, ON, Canada; ^8^ Department of Molecular Genetics, University of Toronto, Toronto, ON, Canada; ^9^ Department of Surgery, University of Toronto, Toronto, ON, Canada; ^10^ Genetics Branch, National Cancer Institute, National Institutes of Health, Bethesda, MD, USA

**Keywords:** miRNA, osteosarcoma, biomarker, plasma, prognosis

## Abstract

Osteosarcoma is the most common malignant bone tumor in children and young adults. Despite the use of surgery and multi-agent chemotherapy, osteosarcoma patients who have a poor response to chemotherapy or develop relapses have a dismal outcome. Identification of biomarkers for active disease may help to monitor tumor burden, detect early relapses, and predict prognosis in these patients. In this study, we examined whether circulating miRNAs can be used as biomarkers in osteosarcoma patients. We performed genome-wide miRNA profiling on a discovery cohort of osteosarcoma and control plasma samples. A total of 56 miRNAs were upregulated and 164 miRNAs were downregulated in osteosarcoma samples when compared to control plasma samples. miR-21, miR-221 and miR-106a were selected for further validation based on their known biological importance. We showed that all three circulating miRNAs were expressed significantly higher in osteosarcoma samples than normal samples in an independent cohort obtained from the Children’s Oncology Group. Furthermore, we demonstrated that miR-21 was expressed significantly higher in osteosarcoma tumors compared with normal bone controls. More importantly, lower expressions of miR-21 and miR-221, but not miR-106a, significantly correlated with a poor outcome. In conclusion, our results indicate that miR-21, miR-221 and miR-106a were elevated in the circulation of osteosarcoma patients, whereas tumor expressions of miR-21 and miR-221 are prognostically significant. Further investigation of these miRNAs may lead to a better prognostic method and potential miRNA therapeutics for osteosarcoma.

## INTRODUCTION

Osteosarcoma (OS) is the most frequent primary malignant bone tumor in pediatric patients comprising of 55% of all bone tumors [1, 2]. Introduction of combination chemotherapy in the 1970s and surgery for localized tumor led to a significant increase in survival rates to 60–70%. However, metastasis is detected in approximately 25% of the patients and the cure rate for patients with metastatic or relapsed disease remains poor (< 20% survival) [3, 4]. Several clinical features are known to be strong prognostic predictors, such as primary metastases, tumor size, complete surgical resection and histologic response to chemotherapy [5]. Further, a retrospective study demonstrated that the time of identification of pulmonary metastases, which is the most prevalent form of metastases in OS, has a significant effect on survival rates. Identification of metastases at the time of diagnosis resulted in a 5-year survival rate of 18% compared to 0% and 6% rates for those identified at the time of preoperative and postoperative chemotherapy [6]. Biomarkers are increasingly used in cancer treatment to refine risk stratification and augment current clinical decision making tools, such as radiographic imaging. Biomarkers can improve or complement the accuracy, sensitivity and specificity of the routinely used detection and imaging methods. For instance, in a recent breast cancer study a circulating 9-miRNA signature accurately predicts recurrence in a patient designated as healthy by mammography [7]. Hence, blood based biomarkers could be useful for monitoring of disease progression and early detection of relapse. Currently no sensitive and specific non-invasive, diagnostic biomarker to distinguish OS from healthy controls exists. Discovery of non-invasive or tumor biomarkers for early detection or prognostication could improve survival of the patients with OS.

miRNAs are small noncoding regulatory RNA molecules, whose main biological function is to increase or decrease the activity of specific mRNAs and ultimately their translation into proteins [8]. Cell-free circulating miRNAs have shown promise as blood-based biomarkers due to their high stability despite endogenous RNAses and long-term storage, as well as ease of isolation and detection [9]. Additionally, they are easily detected in body fluids, consist of simple chemical compositions without complex modifications, are highly conserved between species, and are expressed in a tissue-specific manner [10]. Emerging evidence shows they are dysregulated in cancer, which could provide alternate or additional means of diagnosis, prognosis, and treatment [11]. Using a mouse xenograft model of prostate cancer, a previous study has demonstrated that miRNAs exclusively expressed by human prostate cancer cells are readily detectable in the circulation of xenograft mice, but not in the control mice [9].

Different types of circulating miRNA biomarkers have been discovered in cancers that can be used for monitoring tumor presence/burden or for predicting disease recurrence. There are increasing reports of using either a single miRNA [12] or a signature as a biomarker [13]. For example in breast cancer, circulating miR-10b and miR-373 levels are significantly higher in breast cancer patients with lymph node metastasis compared to both normal and breast cancer patients with no metastasis, indicating that they may be useful as biomarkers for metastases [14]. In gastric cancer, miR-203 levels in serum showed potential to be a predictor of metastases, recurrence and prognosis [15]. Differential expression of five plasma miRNAs (miR-16, miR-25, miR-92a, miR-451 and miR-486-5p) by microarray profiling suggested that they are potential biomarkers for an early stage of gastric cancer [16]. A profile of miR-21, miR-29a, miR-25, miR-200a and miR-486-5p has been identified as a cervical cancer biomarker and miR-29a and miR-200a are associated with the histological grade and the progression stage [17]. All these studies have demonstrated the rapidly growing field of harnessing circulating miRNAs as biomarkers for cancer detection and prognostication.

In addition to circulating miRNAs, tumor miRNAs have also been extensively exploited as cancer biomarkers [18]. For instance, miRNAs have been shown to be diagnostic and prognostic biomarkers in lung cancer [19]. Other genome-wide miRNA profiling or specific miRNA studies have identified both diagnostic and prognostic biomarkers in various cancer types [20–23]. Tumor miRNA biomarkers have also been shown to differentiate two types of skin cancer, i.e. basal cell carcinoma and Merkel cell carcinoma [24], distinguish brain-metastasizing melanoma from non-brain metastasizing tumors [25] and predict chemotherapy response in primary lung adenocarcinoma tissues [26]. In this study, our main goal is to perform an unbiased genome-wide discovery and validation of circulating miRNAs that are associated with OS patients at the time of diagnosis. Our results show that three miRNAs (miR-21, miR-221, and miR-106a) are significantly overexpressed in OS plasma compared to control samples and could be used as non-invasive biomarkers for OS. miR-21 was also significantly overexpressed in OS tumor samples, and tumor expression of miR-21 and miR-221 correlated with prognosis. Our results demonstrate that these miRNAs may be useful in disease detection and monitoring, as well as prognostication, of OS.

## RESULTS

### Genome-wide miRNA profiling identifies candidate circulating miRNAs in OS

**Figure 1:**
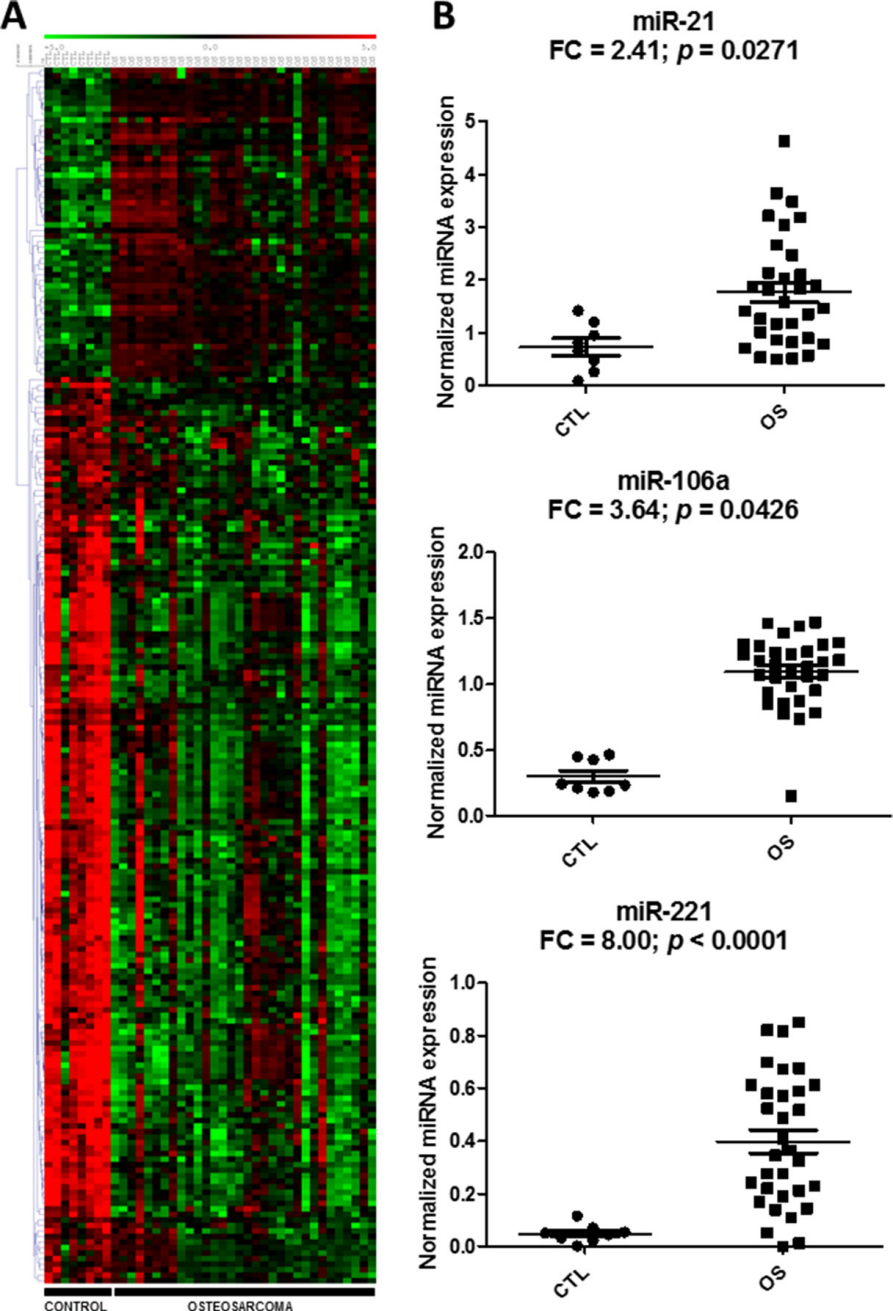
Differential expression of circulating miRNAs in the discovery cohort Heatmap and hierarchical clustering of the expressions (-dCq) of the 220 differentially expressed miRNAs in osteosarcoma (OS) and control samples (**A**). Red and green denote high and low expression, respectively. Scatter plots of the three miRNA candidates indicate relative miRNA expression, which is expressed as normalized linear expression values (2^-dCq^) of each individual miRNA in the OS and control plasma samples (**B**). FC denotes fold change of OS/control samples.

To identify circulating biomarkers that were associated with OS, we analyzed the abundance levels of 752 miRNAs in a discovery set of OS plasma samples (*n* = 32) versus normal plasma samples from healthy donors and children with noncancerous diseases (*n* = 8) using a locked nucleic acid (LNA)-based quantitative reverse transcription-polymerase chain reaction (qRT-PCR) platform. The results showed 220 plasma miRNAs differentially expressed between OS and healthy controls (56 upregulated and 164 downregulated in OS) (Figure 1A, Supplementary Table 5). Because of the large amount of differentially expressed miRNAs, we further screened for the most biologically relevant candidates using the following criteria: (1) up-regulated in OS cell lines [27]; (2) involved in tumorigenesis [28–30], and (3) present as circulating miRNAs in cancer [31, 32]. Based on these additional criteria, three miRNAs (miRNA-21, miR-221, and miRNA-106a), which were expressed significantly higher in OS relative to the normal controls, were selected for further evaluation (Figure 1B). Continuous Cox Proportional Hazard (COXPH) analysis of these three circulating miRNAs indicated that none of them were significantly associated with overall (miR-21: *p* = 0.397; miR-221: *p* = 0.320 and miR-106a: *p* = 0.724) or event-free survival (miR-21: *p* = 0.387; miR-221: *p* = 0.157 and miR-106a: *p* = 0.943) in our series.

### Validation of the three candidate miRNAs in an independent plasma cohort

**Figure 2:**
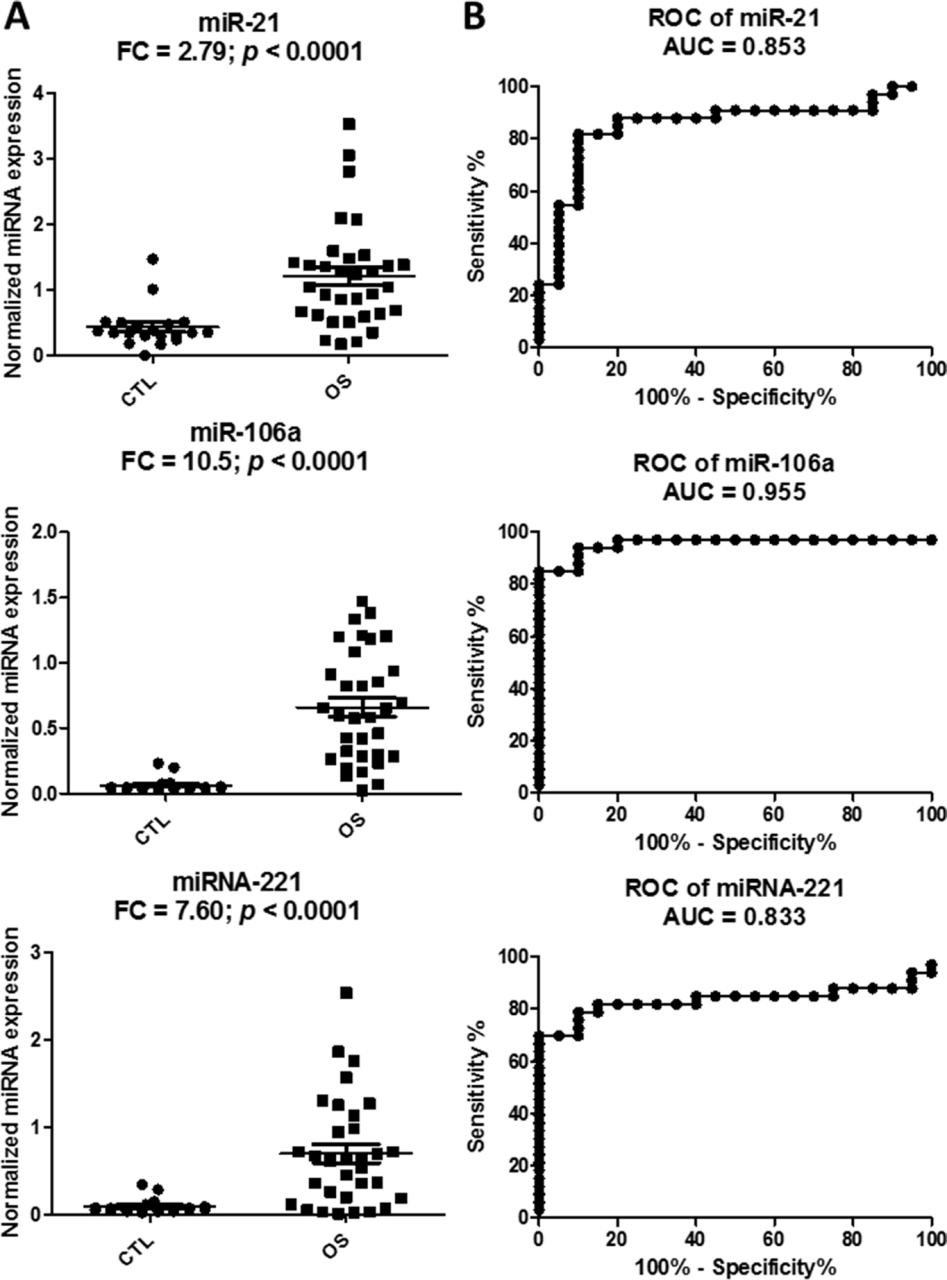
Validation of the three miRNA candidates in the plasma samples of an independent cohort from the Children’s Oncology Group Scatter plots to show higher miRNA expression levels of miR-21, miR-221 and miR-106a in the osteosarcoma (OS) plasma samples relative to the control samples of the validation set (**A**). Relative miRNA expression is expressed as normalized linear expression values (2^-dCq^). Receiver Operating Characteristics analysis of the three miRNAs in discriminating the OS cases from the control cases (**B**). FC denotes fold change of OS/control samples.

To validate the higher levels of the three circulating miRNAs in OS patients when compared to normal subjects, LNA-based qRT-PCR was performed on an independent cohort of human plasma samples obtained from the Children’s Oncology Group (*n* = 29) and healthy donor controls (*n* = 17). The results demonstrated that all the three selected miRNAs were expressed significantly higher (*p* < 0.05 and fold change (FC) > 2-fold) in OS samples when compared to normal samples (Figure 2A). Furthermore, the receiver operator curve (ROC) analysis showed that all three circulating miRNAs had very good diagnostic characteristics with AUC values > 0.8, with miR-106a being the best (Figure 2B).

### Tumor expression of three miRNAs in OS

**Figure 3:**
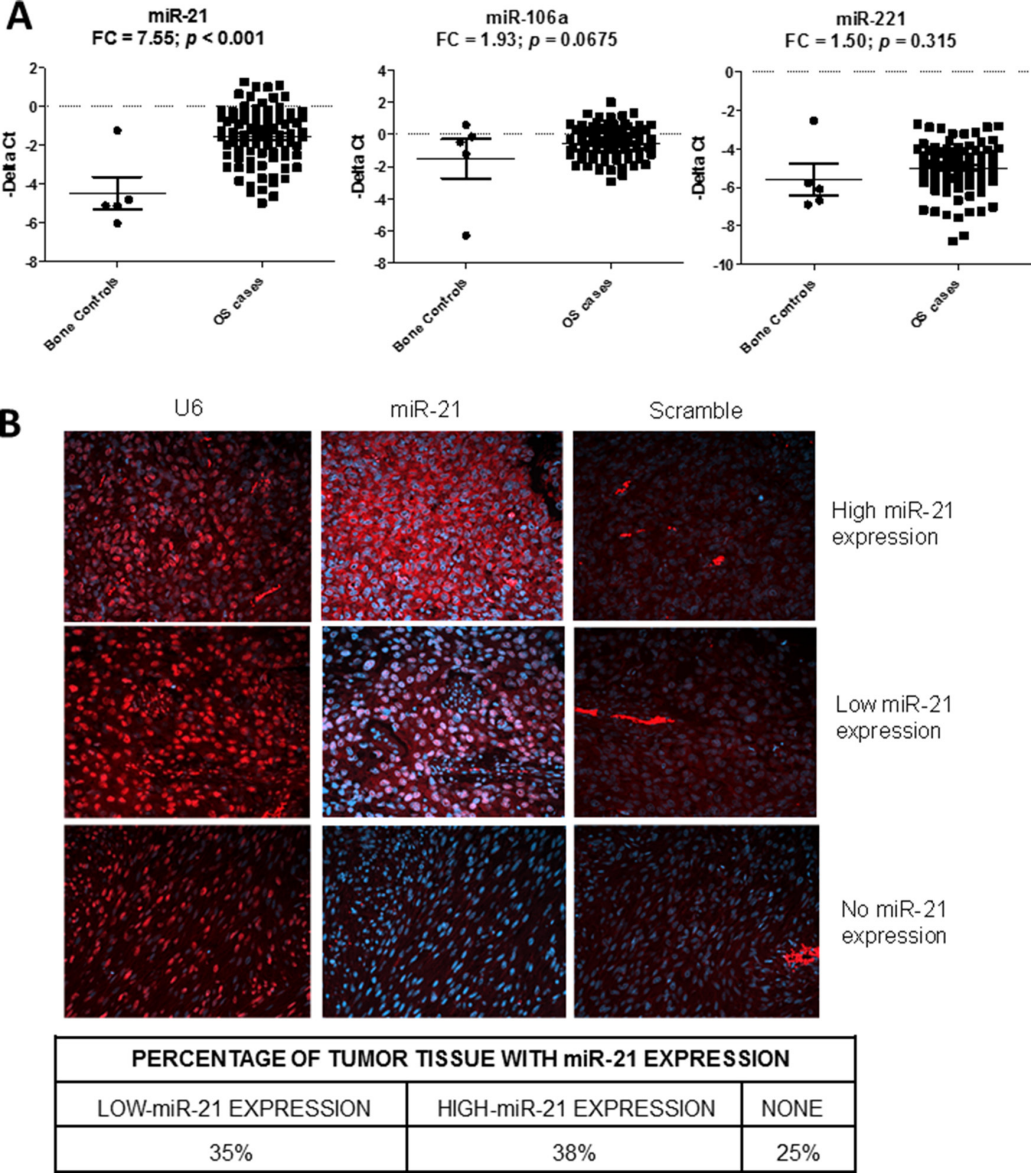
Detection of the miR-21, miR-106a and miR-221 in osteosarcoma (OS) tissues Scatter plots showing the tumor expression (-dCt) of miR-21, miR-106a and miR-221 in osteosarcoma tumor (*n* = 89) and normal bone (*n* = 5) samples as measured by qRT-PCR. (**A**) FC denotes fold change of OS/control samples. *In-situ* hybridization (ISH) 20× images showing the miR-21 expression (red) in OS tissues in formalin-fixed, paraffin embedded OS tissue microarray (**B**). The blue color indicates DAPI (4′,6-diamidino-2-phenylindole) stained nuclei. Left, middle and right panels show U6 (positive control), miR-21, and scrambled probe (negative control) images, respectively.

Since the three miRNAs were elevated in the peripheral blood samples of OS patients, we tested if these circulating miRNAs were also overexpressed in OS tumor tissues. We compared the expressions of the miRNAs in 89 OS tumor tissue samples obtained from our TARGET initiative with five normal bone controls, which included two samples of fetal normal human bone (FNB), two samples of normal human bone (NB), and one sample of normal human osteoblast (NHOst). The analysis showed that only miR-21 was significantly overexpressed in the tumor tissues (*p* < 0.001, FC = 7.56) (Figure 3A). To confirm if miR-21 was expressed in OS tumor tissues, we examined the miR-21 expression in an OS tissue microarray (TMA) using *in-situ* hybridization (Figure 3B). U6 and scrambled probes were used as a positive and a negative control, respectively. We tabulated the miR-21 expression in the TMA (Figure 3B) and found that miR-21 was expressed in approximately 70% of the OS tissues.

### Prognostic significance of the three miRNAs

**Figure 4:**
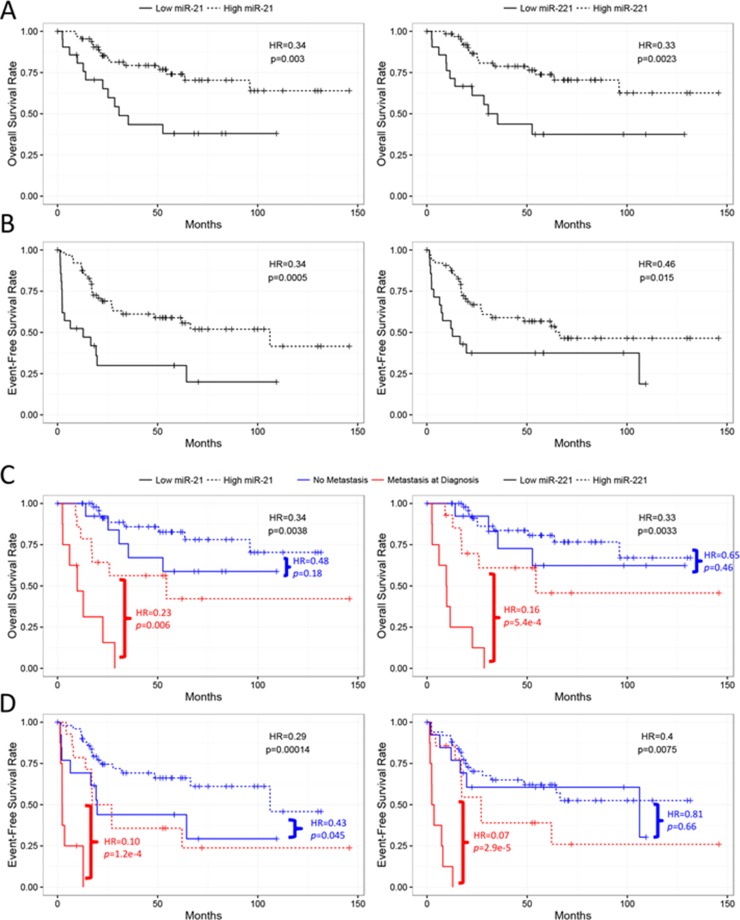
Kaplan-Meier analyses of miR-21 and miR-221 in the TARGET cases miR-21 and miR-221 are significantly associated with overall (**A**) and event-free (**B**) survival when risk-stratified by 1st quartile of –dCt values. miR-21 and miR-221 are further independently prognostic from metastatic disease at diagnosis as well as significant in only the metastatic subpopulation for both overall (**C**) and event-free (**D**) survival. miR-21 alone also significantly stratifies patients presenting with no metastasis at diagnosis (D). All statistics presented were computed by log-rank tests.

**Figure 5:**
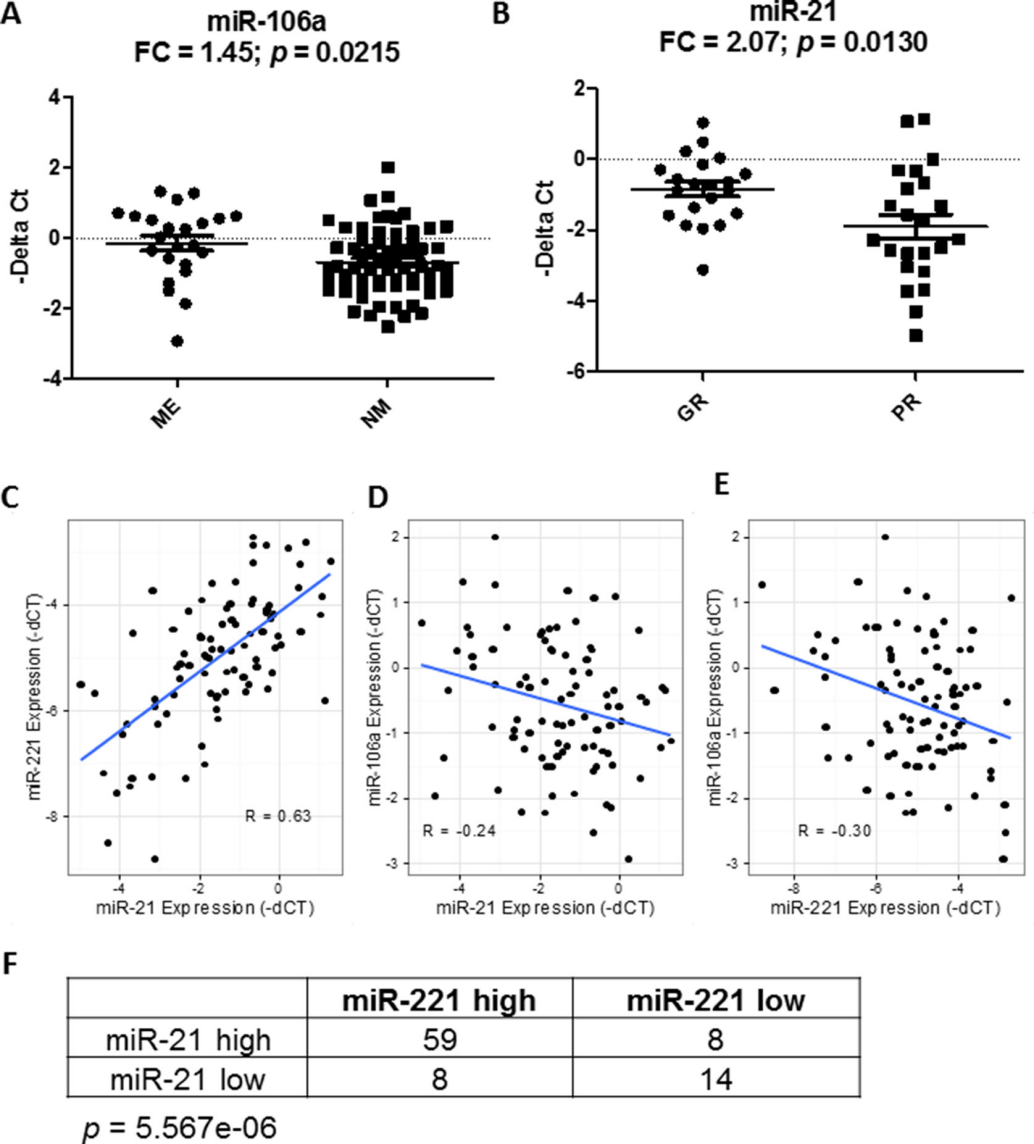
Correlation of miRNAs with known prognostic factors and among themselves The differential expression of miR-106a (**A**) and miR-21 (**B**) with metastasis at diagnosis and histologic response. FC denotes fold change of ME/NM and GR/PR samples. ME, NM, GR and PR denote metastasis, no metastasis, good response and poor response, respectively. The expressions of miR-21 and miR-221 were highly positively correlated to one another in osteosarcoma samples (**C**) whereas miR-106a exhibits negative correlation with both miR-21 (**D**) and miR-221 (**E**). R represents the Pearson correlation coefficient. Comparing the risk-stratified grouping with respect to miR-21 and miR-221 shows that most patients fall into the same risk group, and are statistically significant using Fisher’s exact test (**F**).

Despite only miR-21 showing overexpression in the OS tumors, we tested whether expression of each miRNA correlated with prognosis. Survival analysis showed that lower expression levels of both miR-21 (*p* = 0.0135, HR = 0.71) and miR-221 (*p* = 0.0004, HR = 0.57), but not miR-106a (*p* = 0.07, HR = 1.39), were significantly associated with poor overall survival in the continuous COXPH analysis. Similar results were also observed for event-free survival (Supplementary Table 1). The survival curves of the high and low expression levels of miR-21 and miR-221 using the first quartile as a cutoff displayed a significant separation (Figure 4A and 4B). Since metastasis is a known prognostic factor at diagnosis, we tested if the prognostic significance of miR-21 and miR-221 was dependent on the metastatic status at diagnosis. The result of the stratified analysis indicated that the two miRNAs remained significant for both overall and event-free survival after controlling for metastasis at diagnosis, suggesting that the miRNAs were independent prognostic factors (Figure 4C and 4D, Supplementary Table 2). When considering only patients with metastasis at diagnosis, similar results for miR-21 and miR-221 further indicated that lower expression of the two miRNAs could identify an extremely high-risk subpopulation even among the already high-risk, metastatic patients (Figure 4C and 4D). In contrast, only miR-21 was significantly associated with event-free survival in patients with localized disease at diagnosis (Figure 4D). Consistent with the stratified analysis results, neither miR-21 nor miR-221 were significantly associated with metastasis at diagnosis. However, miR-106a expression significantly correlated with metastatic status (*p* = 0.035, μ_met_ = −0.15, μ_no_met_ = −0.69, Figure 5A and Supplementary Figure 1A), even though it was not prognostically significant.

Next, we tested if the prognostic significance of miR-21 and miR-221 was independent from another known prognostic factor in OS, i.e. histologic response to adjuvant chemotherapy. However, the histologic response can only be measured after the adjuvant chemotherapy is completed. The stratified analysis showed that none of the miRNAs were significant after controlling for the histologic response, indicating that they were not prognostically independent (Supplementary Table 3). In addition, miR-21 expression, but neither miR-221 nor miR-106a, was significantly associated with histologic response (*p* = 0.012, μ_poor_ = −1.90, μ_good_ = −0.69, Figure 5B and Supplementary Figure 1B). Together, the results suggest that the prognostic significance of miR-21 and miR-221 may be in part explained by their association with the histologic response, and miR-21 may be used as a predictive biomarker in OS to identify patients who are likely to respond poorly to chemotherapy at diagnosis.

Lastly, the correlation analysis showed that miR-21 and miR-221 were significantly correlated with each other (R = 0.63, *p* = 3.1e-11), while both were significantly negatively correlated with miR-106a (miR-21-miR106a: R = −0.24, *p* = 0.02; miR-221-miR-106a: R = −0.30, *p* = 0.005, Figure 5C–5E). The high-risk and low-risk groups defined by miR-21 and miR-221 were significantly correlated (Fisher’s exact test, *p* = 5.567e-5, Figure 5F). Also, only miR-221 remained significant in a multivariate analysis of all three miRNAs for overall survival (*p* = 0.016), and close to significant for event-free survival (*p* = 0.08) (Supplementary Table 4). Among the three miRNAs, the analysis suggested that miR-221 may be the most promising prognostic biomarker for further evaluation.

## DISCUSSION

Development of metastases or disease relapse is a major cause of mortality in OS. Identification of non-invasive and easy-to-use biomarkers for monitoring tumor burden or early detection of relapse has the potential to augment the clinical care of patients with OS. Extracellular miRNAs can be detected in various biofluids, including whole blood, plasma and serum. Circulating miRNAs are stable in these body fluids by binding to proteins or enclosed in vesicles, rendering them to be a good source for biomarker studies in various diseases [33, 34]. Based on published circulating miRNA studies in OS, we found that serum samples were more frequently used than plasma. Although whole blood samples have also been used in other cancers like breast cancer [35], it has not been reported in OS. Notably, comparison of miRNA expression levels between serum and plasma samples by previous studies have shown that the miRNA levels in these two types of blood samples are highly correlated [36]. However, another study has shown that specific miRNAs may not consistently be detected among serum, plasma and whole blood, such as miR-504 and miR-138. Caution should be exercised in selecting the appropriate sample type if detection of specific circulating miRNAs is required [37].

In this study, we identified and subsequently validated three miRNAs, miR-21, miR-106a and miR-221, which were elevated in the peripheral blood samples of OS patients when compared to healthy controls. Our findings are corroborated by other studies showing that these miRNAs are upregulated in the circulation of OS patients [38, 39]. We further demonstrated that these three miRNAs have high AUC values, suggesting they have good discriminatory power to distinguish OS patients from normal subjects, which could be used as non-invasive biomarkers for monitoring relapses and tumor burden in OS. Further investigations of longitudinal blood samples collected from different stages during the course of therapy, e.g. after definitive surgery and before and after relapses, are warranted to determine the clinical utility of these biomarkers for disease monitoring and early detection of relapses. In OS, a higher level of circulating miR-21 has been shown to correlate with initial metastasis, a poor tumor response to neoadjuvant chemotherapy and a reduced overall survival rate [40]. In addition, circulating miR-221 in OS has been shown to be a diagnostic biomarker and a prognostic biomarker for recurrence-free and overall survival [39]. Although our results confirmed that these two circulating miRNAs were elevated in OS plasma, no prognostic significance was found in our cohort. This discrepancy may be due to the relatively small sample size used in our discovery study, sample collection biases, and patient heterogeneity. A more extensive prognostic analysis of circulating miR-21 and miR-221 in a larger and adequately powered cohort will be needed to better determine their prognostic significance in OS.

In this study, we have reported an unbiased and genome-wide profiling of circulating miRNAs in OS patients and controls. These results allow us to compare our results with previous circulating miRNA biomarker findings in OS. Although most of the circulating miRNA studies in OS have involved specific preselected miRNAs, three studies have used miRNA profiling [41–43]. One miRNA profiling study identified a diagnostic biomarker panel of 4 upregulated miRNAs (miR-195–5p, miR-199a-3p, miR-320a, and miR-374a-5p), which are significantly reduced in postoperative samples [42]. Corroborated by our findings, they also found that miR-21, miR-199a-3p, miR-107, miR-335 and miR-374a-5p are upregulated in OS plasma. Another miRNA profiling study identified a higher level of circulating miR-199a-5p in OS patients, which is consistent with our profiling results [43]. However, they did not observe higher levels of circulating miR-21, miR-221 or miR-106a in OS patients. Finally, Li *et al.* found that circulating miR-106a is downregulated in OS, which is contradictory to our finding [41]. The discrepancies among these previous studies and our study may be due to different experimental designs, such as the use of pooled samples, U6 as a normalizer, and small sample sizes. The use of different biological samples, such as serum vs. plasma, and control samples may also contribute to the variations among these studies.

In the preselected miRNA studies, circulating miR-21 and miR-221 were shown to be expressed higher in OS, which are consistent with our results [38–40]. Recently, miR-223 and miR-148a were identified as diagnostic and prognostic biomarkers and miR-34b was found to be associated with clinical risk in OS [44–46], but their results were not confirmed in our profiling data. In summary, both similarities and dissimilarities are found when we compare our study with the previous miRNA studies in OS. This may be due to the use of different normalization methods, patient cohorts, and miRNA quantitation methodologies in different studies. Standardization of the normalization method and increase of the sample size in future circulating miRNA studies will help to validate the diagnostic and prognostic significance of the biomarker candidates identified in these studies.

In addition to the circulating biomarkers, we also showed that miR-21 is upregulated in OS tumor tissues relative to bone controls, which is consistent with previous studies [47]. Since there are no widely accepted bone controls in the field, we used fetal and adult bone cells as well as normal osteoblasts as the controls in this study. Nonetheless, because of the small sample size (*n* = 5) and heterogeneous nature of the bone controls, the lack of differential expression of miR-221 and miR-106a in OS vs. controls needs to be interpreted with caution. More interestingly, we showed that lower expressions of miR-21 and miR-221 in OS tumors are associated with poor overall and event-free survival. Nonetheless, Ren *et al.* described that higher miR-21 expression predicts poor overall and disease-free survival in OS [48]. These contradictory findings may be explained by differences in race (multiracial in our study vs. single race in their study), age (97% of patient population less than 25 years old in our study compared to 63% in their study), miRNA quantification procedures, and treatment regimens in respective cohorts. To our knowledge, this is the first study to report the prognostic significance of tumor miR-221 in OS.

Furthermore, our correlation and multivariate survival analysis results further suggest that the tumor expressions of the two miRNAs, miR-21 and miR-221, are highly correlated, with miR-221 remaining the most significant prognostic biomarker after adjusting for the other two miRNAs in OS. Our analysis result also showed that miR-21 and miR-221 were not independent prognostic factors from the histologic response. miR-21 significantly correlated with histologic response, which can only be measured after the completion of adjuvant chemotherapy. Attempts to modify postoperative chemotherapy have not yielded any significant survival benefit, suggesting that an early predictive biomarker before the treatment initiation would be clinically preferable [49]. Upon further validation, miR-221 and miR-21 may be used as a novel prognostic and predictive biomarkers, respectively, at the time of diagnosis, so that alternative or more aggressive therapies could be offered up front, if available, to improve their survival.

miR-21 has been found to be upregulated across six major types of cancers [50] and considered to be an oncogene [29, 51]. It has been shown to promote cancer development and progression [52], inhibit tumor suppressor genes [53–55], and regulate chemosensitivity [56, 57]. As a biomarker in OS, circulating miR-21 has been shown to be upregulated in OS serum [40], plasma [38] and tumors [47, 48]. One of these studies further showed that a higher level of circulating miR-21 correlates with advanced Enneking stage, poor tumor response to neoadjuvant chemotherapy and a reduced overall survival rate in OS [40]. Functionally, miR-21 has been shown to promote metastatic potential through modulation of the tumor suppressor protein RECK expression in an OS cell line [47]. It can also enhance proliferation, invasion and inhibit apoptosis through PTEN/PI3K pathway in OS cells [58]. Inversely, p16 INK4A (a CDK inhibitor) decreases the migratory and invasive capabilities of OS cells through miR-21 downregulation [59]. Thus, it was puzzling why lower expression of tumor miR-21 correlated with a poor histologic response and a poor outcome in our study. However, most of these functional studies are *in vitro* studies with OS cell lines, the *in vivo* function of miR-21 in the tumor is still unclear. Nevertheless, lower miR-21 expression significantly correlates with poor survival in some cancers, including non-small lung carcinoma [60], diffuse large B cell lymphoma [61], and colorectal cancer [62], suggesting that miR-21 can also act as a positive prognostic factor. More importantly, OS is prone to chemoresistance and a recent study has revealed that lower miR-21 levels lead to increased cisplatin resistance in OS [63]. Hence, we postulate that lower expression of miR-21 may increase cisplatin resistance in OS, which leads to poor histologic response and survival of the patients. However, further validation of the prognostic significance of miR-21 in OS patients and *in vivo* analysis of its function in a mouse model of OS will be needed to shed light on the role of miR-21 in OS.

Similar to the previous studies in prostate cancer [64, 65] and breast cancer [66], our results showed that lower miR-221 expression in OS tumors correlated with poor prognosis. However, other studies showed that higher miR-221 expression correlates with poor prognosis in solid tumors, including colon cancer [20], non-small cell lung cancer [23], and glioma [67]. The miR-221 has important biological roles in regulating tumor progression, tumorigenesis, stem cell phenotype, chemoresistance, tumor cell proliferation and radioresistance [30, 68–71]. The known downstream targets of miR-221 include HDAC6 [68], DNMT3b [70], NOSTRIN [72], E-cadherin [73], uPAR7b [74], PTEN [75] and the tumor suppressor proteins p27 and p57 [76]. Interestingly, we have recently reported that p27 (KIP1 and CDKN1B), a known direct target of miR-221 [76], is frequently mislocalized to the cytoplasm of OS tumors, which increases tumor cell migration and invasion and metastasis in OS [77]. Our working model is that low expression of miR-221 may lead to overexpression of p27, which is exported from the nucleus to the cytoplasm. The overexpression and mislocalization of p27 promote tumor progression and metastasis, thus leading to a poor outcome in OS patients. This finding led us to further postulate that replacement of miR-221 expression in high-risk OS cases may lead to downregulation of cytoplasmic p27 and decrease the metastatic potential of OS cells. To support this notion, we have previously demonstrated that silencing p27 expression in OS cells harboring cytoplasmic p27 can lead to lower motility and invasiveness [77]. miRNA replacement therapy is an emerging and promising field in cancer therapy, which has recently attracted a lot of interest. Clinical trials are currently underway to evaluate the safety and efficacy of miRNA therapeutics, such as the use of MRX34 encapsulated in a liposomal nanoparticle in cancer treatment [78]. A similar strategy could be explored to evaluate the therapeutic effect of miR-221 in OS.

miR-106a was one of the first miRNAs shown to be overexpressed in multiple solid tumor types by a large scale miRNAome analysis [50]. In OS, the role of miR-106a is still unclear. miR-106a has been found to be overexpressed in OS cell lines when compared to normal bones [27], and in OS tumors when compared to both human osteoblasts and mesenchymal stem cells [79], while lower miR-106a expression in OS has also been reported [80]. In this study, we did not find that miR-106a was significantly upregulated or downregulated in OS tumors when compared to the normal bone controls. The increase of miR-106a in the plasma of OS patients may be derived from other tissues in the body rather than the tumor itself.

Contrary to previous studies of circulating miRNAs in OS, we have reported genome-wide profiling of circulating miRNAs in OS patients and controls. Our study has also addressed some of the weaknesses in earlier OS studies with miRNA profiling, such as the use of pooled samples, questionable control (U6), and small sample size [41–43]. Despite the differences in methodology, upregulation of circulating miR-21, miR-221, and miR-106a in OS has been observed in previous studies [38–40, 42]. Nevertheless, this study had its own limitations. Due to the lack of matched tissue and plasma samples, the correlation between tumor and blood expression of the three miRNAs could not be properly evaluated. Hence, the question whether the circulating miRNAs are derived or released from tumor cells is still unclear. Another limitation is that longitudinal samples were not available to evaluate the utility of the circulating miRNAs in monitoring tumor burden and relapses.

In summary, our current study has identified and validated the elevated levels of circulating miR-21, miR-221 and miR-106a in OS, suggesting that they could be potentially used as non-invasive biomarkers for detecting OS, monitoring disease burden, and/or detecting early relapse. We have further demonstrated that miR-21 is upregulated in OS tumors and the expressions of miR-21 and miR-221 are prognostically significant in a large cohort of OS patients. Functional studies with miRNA mimics to further dissect the anti-tumor roles of miR-21 and miR-221 in OS may lead to the development of novel miRNA-based therapeutics to improve the survival of OS patients with a poor prognosis.

## MATERIALS AND METHODS

### Patient samples and characteristics

To conduct genome-wide miRNA profiling to discover biomarker candidates, plasma samples from 32 OS patients were used. The samples were collected at the time of diagnosis from the Texas Children’s Hospital (TCH) and other collaborating institutions. The patient characteristics are summarized in Supplemental Table 6 [81]. All patients gave consent to institutional review board-approved protocols. The patient samples were compared with normal plasma samples (*n* = 8). Four of the normal samples were obtained from Equitech Enterprises, Inc. (Kerrville, TX) and the rest from anonymized patients with noncancerous diseases (*n* = 4), i.e. child checkup, flu, constipation, gastroenteritis, or febrile seizure. To validate the three miRNA candidates, OS plasma samples (*n* = 29) were obtained from the Children’s Oncology Group (Protocol H-6650 and H-31361). The patient samples were compared with control plasma samples from 18 year-old healthy individuals (*n* = 17) from Bioreclamation LLC (Hicksville, NY). The patient characteristics of the validation cohort are summarized in Supplementary Table 6 [82].

### Plasma sample processing

All the plasma samples were collected in EDTA-containing tubes and the plasma supernatant was collected after centrifugation in their respective standard protocols, e.g. 1,000 rpm for 10 min at RT for the TCH samples, and stored in aliquots at −80^°^C until use.

### miRNA profiling in plasma

Total RNAs, including small RNAs (miRNA), were isolated from plasma samples (50 µL) using the miRNeasy Mini Kit according to manufacturer’s protocol (Qiagen, Inc., Valencia, CA). Each reverse transcription (RT) reaction consisted of 72 µL RT master mix from miRCURY universal cDNA synthesis kit (Exiqon, Inc., Woburn, MA) and 8 µL of RNA to generate 80 µL cDNA. The cDNA of each plasma sample was then diluted to 4.4 mL with water and then combined with 4.4 mL of PCR SYBR green master mix (Exiqon) and miRNA expression profiling was carried out using the miRCURY LNA™ Universal RT miRNA PCR, Ready-to-Use Human Panel I V2 (Exiqon). Two of these panels were run for each plasma sample. The PCR reactions were performed on a LightCycler 480 (Roche Diagnostics Corp, Indianapolis, IN) to generate the raw Cq value for each of the miRNA in the patient and control samples.

### miRNA assays to validate miR-21, miR-221, and miR-106a

Each plasma sample (250 µL) was filtered using a 0.22 µm filter of which 200 µL was used for RNA extraction. Qiazol lysis reagent was used for sample denaturation. Carrier RNA from the bacteriophage MS2 (0.625 ng) was added to minimize the loss of small RNA molecules followed by chloroform (50 µL) for separation of miRNAs. miRNeasy silica spin columns from Qiagen were used to isolate miRNAs as per manufacturer’s instructions and were eluted in a 50 µL volume of nuclease free water and stored at −80°C. miRCURY universal cDNA synthesis kit was used for first strand cDNA synthesis from 1 µL of RNA. RT reactions were done on a PTC-100 thermocycler (Bio-Rad Laboratories, Inc., Hercules, CA) and samples were stored at −20°C. The spike-in synthetic RNAs, UniSp2 and UniSp6 (Exiqon) were used to monitor the efficiency of the RNA extraction and cDNA synthesis, respectively. Four microliters of cDNA (diluted 1:20 in nuclease free water) were used in qPCR reactions done in duplicates in a final volume of 10 µL consisting of 5 µL of PCR SYBR green master mix and 1 µL of specific PCR primer (Exiqon) on ABI StepOnePlus real time PCR system (ThermoFisher Scientific, Waltham, MA). The method has been previously described in [82].

### miRNA *in-situ* hybridization (ISH)

miRCURY LNA miRNA ISH optimization kit was used for *in situ* hybridization of miRNA in formalin-fixed and paraffin-embedded OS tissue microarrays (TMA, Imgenex Corp, Littleton, CO). The clinical characteristics of the OS cases in the TMA are described in Supplementary Table 7. The TMA’s were first deparaffinized in xylene and rehydrated with an ethanol gradient. Proteinase K unmasking was done by treating with 20 μg/mL Proteinase K (Exiqon) for 10 min at 37°C. The TMAs were fixed with 4% formaldehyde in PBS followed by EDC (1-Ethyl-3-(3-dimethylaminopropyl carbodiimide). miR-21, U6, and negative control (scrambled miRNA) probes with LNA-modified and 5′- and 3′-DIG-labeled oligonucleotides (Exiqon) were tested in each experiment. They were prepared in the hybridization buffer containing yeast tRNA, formamide, heparin, and 0.1 % Tween 20 and incubated with the tissue spots for 1 hour covered with HybriSlip (Electron Microscopy Sciences, Hatfield, PA). After a couple of washes in Saline Sodium Citrate buffer, the TMA’s were blocked in BSA/PBS for 30 minutes at RT followed by incubation with anti-Digoxigenin-POD Fab fragments (Roche Diagnostics) for 1 hour at RT. The slides were washed with PBST and followed by Cy5 tyramide detection (TSA-Plus cyanine 5 System, PerkinElmer, Inc, Waltham, MA). Slides were then mounted with Prolong gold antifade reagent with DAPI (ThermoFisher Scientific). The signals and images of the probes were detected and captured using Nikon Eclipse E800 with a 20× magnification.

### Data analysis of plasma miRNAs

In the miRNA profiling analysis, raw miRNA Cq values were normalized by the sum of miR-320a and miR-15a-5p expression values to derive dCq values similar to previously described [82], and the negative dCq values were used as normalized miRNA expressions. miRNAs with missing values were filtered, resulting totally 313 miRNAs for the statistical analysis. Differential expression analysis was performed using 2-sample *t*-test with confidence level of 90% and false discoveries of 5% as implemented in BRB Arraytools [83]. The significant miRNAs were used to construct the heatmap with hierarchical clustering of Euclidean distance and average linkage using Multi-Experiment Viewer (MeV) [84]. The expression values (Cq) were mean-normalized by row. The ROC graphs were generated by Prism (GraphPad software, Inc, La Jolla, CA). For the validation analysis, a sum of miR-320a, miR-15a-5p and UniSp2 were used for normalization [82]. Differential expression was performed using a two-sample, two-tailed *t*-test comparing the 2^−dCq^ values of the two groups (*p* < 0.05).

### Data analysis of tumor miRNAs

To determine whether the tumor expression of the three miRNAs identified in blood analysis were also differentially expressed and prognostically significant in tumor samples, we analyzed the miRNA data generated from 89 OS tumors from the NCI’s TARGET consortium. The data are available for download at https://ocg.cancer.gov/programs/target/using-target-data. The normal controls consisted of two fetal bone samples and two adult bone samples, and the normal human osteoblasts (NHOst) were purchased from the BioChain Institute (Newark, CA). RNAs were extracted from samples using a modification of the RNA AllPrep kit (Qiagen Inc, Valencia, CA). The flow-through from the Qiagen DNA column was processed using a mirVana miRNA Isolation Kit (Ambion, Foster City, CA). The TaqMan Low Density Array (TLDA) Human MicroRNA Panel (Applied Biosystems, Inc., Foster City, CA) platform was employed for miRNA profiling. All experimental procedures and conditions were conducted according to manufacturer’s protocols. ΔCt values for each miRNA were computed relative to U6 for each card. Missing values were imputed using the *imputation* package (k = 10). The negative ΔCt values of miR-21, miR-221, and miR-106a were used for the statistical analyses. Detailed description of the data and pre-processing methods is published elsewhere. Correlation with clinical covariates, such as metastasis status at diagnosis and histologic response, was analyzed using 2-sample, unpaired *t*-tests. Expression was further analyzed with respect to overall and event-free survival using continuous Cox Proportional Hazards models (COXPH). Kaplan-Meier curves were generated using first quartile expression value for each miRNA as a cutoff. Stratified analyses were performed with the miRNA expression and clinical covariates. Significance was determined using the two-tailed log-rank statistic for all survival analyses. 5-year survival estimates for all Kaplan-Meier curves are provided with 95% confidence intervals. *p* < 0.05 was considered significant in all statistical tests. Metastasis at diagnosis refers to whether a patient had presence of any detectable metastatic lesions at the time of diagnosis, whereas histologic response refers to greater than (“good” response) or less than (“poor” response) 90% tumor cell necrosis from preoperative chemotherapy observed after definitive surgery. HR refers to “hazard ratio”.

## SUPPLEMENTARY MATERIALS FIGURE AND TABLES


