# Supplementary material for: Biomarker significance of plasma and tumor miR-21, miR-221, and miR-106a in osteosarcoma
Source: Oncotarget. 2017 May 27;8(57):96738–52. doi: 10.18632/oncotarget.18236 (PMC5722519; doi:10.18632/oncotarget.18236)
Supplement: Supplementary file 1 [file oncotarget-08-96738-s001.pdf]

Biomarker significance of plasma and tumor miR-21, miR-221, and miR-106a in osteosarcoma

Supplementary Materials

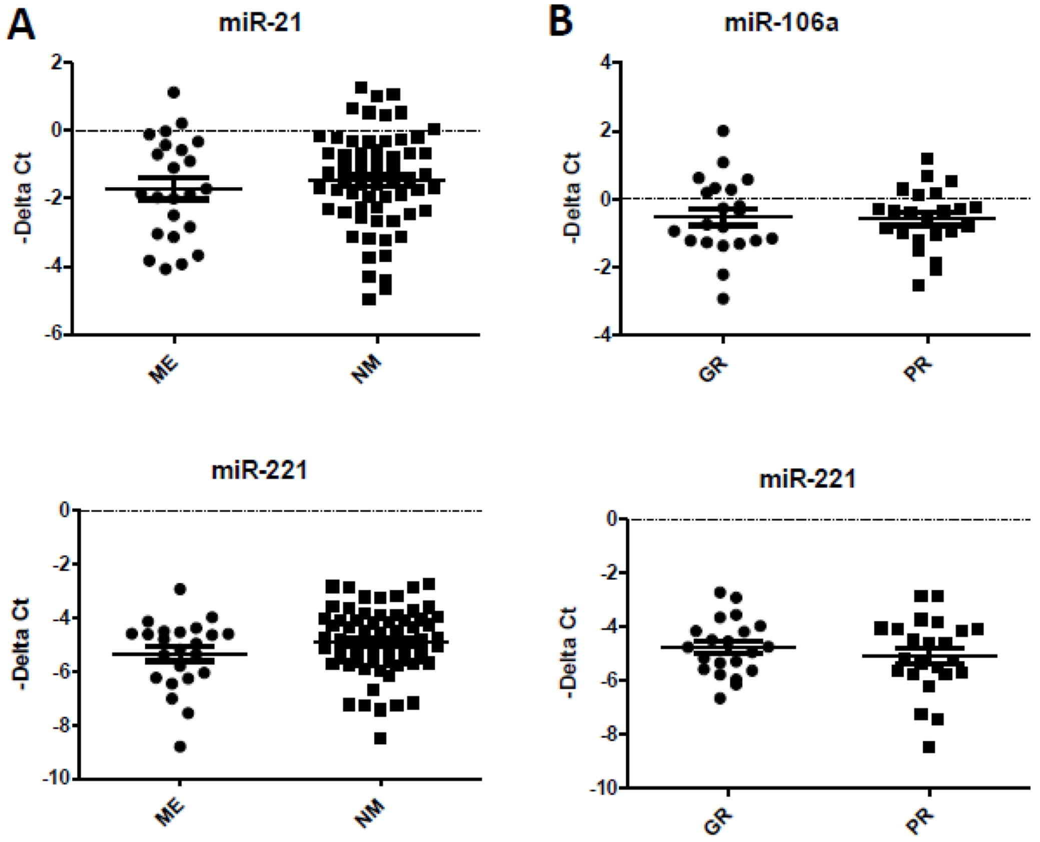

Supplementary Figure 1: Scatter plots show the expression of miR-21 and miR-221 in the comparison of metastatic (ME) and non-metastatic (NM) cases (**A**), and miR-106a and miR-221 in the comparsion of good (GR) and poor (PR) responders (**B**). No statistical significance ($p < 0.05$) was found in the analyses.

Supplementary Table 1: Low expressions of tumor miR-21 and miR-221 significantly correlate with both overall and event-free survival in continuous COXPH

Survival	miRNA	Hazard Ratio	*p* value	Survival rate (High expression)	Survival rate (Low expression)
Overall					
	miR-21	0.706	0.014	74% (95% CI, 63%–87%)	38% (95% CI, 21%–68%)
	miR-221	0.574	0.000	74% (95% CI, 63%–87%)	37% (95% CI, 21%–68%)
	miR-106a	1.396	0.07	61% (95% CI, 49%–75%)	78% (95% CI, 60%–100%)
Event-free					
	miR-21	0.741	0.008	59% (95% CI, 47%–73%)	30% (95% CI, 15%–60%)
	miR-221	0.676	0.002	57% (95% CI, 45%–71%)	37% (95% CI, 21%–66%)
	miR-106a	1.232	0.176	47% (95% CI, 36%–62%)	68% (95% CI, 50%–93%)

Supplementary Table 2: Stratified COXPH analysis results show that miR-21 and miR-221 remain to be significant in both overall and event-free survival after controlling for metastasis at diagnosis, indicating these two miRNAs were independent prognostic factors

Survival	miRNA	Hazard Ratio	*p* value
Overall			
	miR-21	0.691	0.012
	miR-221	0.540	< 0.001
	miR-106a	1.297	0.177
Event-free			
	miR-21	0.691	0.002
	miR-221	0.646	0.001
	miR-106a	1.185	0.284

Supplementary Table 3: Stratified COXPH analysis results show that none of the miRNAs remain significant after controlling for the histologic response

Survival	miRNA	Continuous Hazard Ratio	Continuous *p* value
Overall			
	miR-21	0.986	0.945
	miR-221	0.733	0.139
	miR-106a	0.908	0.706
Event-free			
	miR-21	0.931	0.648
	miR-221	0.791	0.213
	miR-106a	0.867	0.533

Supplementary Table 4: miR-221 remains significant after a multivariate COXPH analysis with all three miRNAs

Survival	miRNA	Hazard Ratio	*p* value
Overall			
	miR-21	1.015	0.940
	miR-221	1.660	0.016
	miR-106a	0.804	0.281
Event-free			
	miR-21	1.133	0.413
	miR-221	1.335	0.080
	miR-106a	0.905	0.539

Supplementary Table 5: Differential expression of the 220 miRNAs in the plasma miRNA profiling experiment

		**Significant miRNA in OS vs. controls**					
**Upregulated in OS**		**Downregulated in OS**					
miR-199a-5p	miR-543	miR-502-3p	miR-363	miR-877*	miR-491-3p	miR-125b-1*	miR-892a
miR-1974	miR-18b	miR-545	miR-188-3p	miR-331-5p	miR-603	miR-30c-2*	miR-200a*
miR-199a-3p	miR-15b	miR-574-3p	miR-29b-1*	miR-181a*	miR-720	miR-485-3p	miR-551b*
miR-423-3p	miR-145	miR-433	miR-127-5p	miR-1245	miR-100	miR-520h	miR-675b
miR-18a	miR-107	miR-30a*	miR-550*	miR-1179	miR-1260	miR-1266	miR-518f*
miR-376c	miR-103	miR-181c	miR-196b*	miR-662	miR-708*	miR-548h	miR-541*
miR-151-5p	miR-191	miR-378	miR-654-5p	miR-92b*	miR-218-1*	miR-888	miR-182*
miR-148b	miR-26b	let-7b*	miR-496	miR-1254	miR-937	miR-1471	miR-450b-3p
miR-766	miR-335	miR-758	miR-122	miR-765	miR-589	miR-885-3p	miR-1183
miR-221	miR-425	miR-491-5p	miR-31	miR-675*	miR-555	miR-616	
miR-328	miR-103	miR-598	miR-940	miR-628-5p	miR-558	miR-637	
miR-130a	miR-20a	miR-125b	miR-503	miR-643	miR-596	miR-346	
miR-326	miR-19b	miR-744*	miR-582-3p	miR-638	miR-1914	miR-518d-5p	
miR-33a	miR-21	miR-99b*	miR-665	miR-760	miR-1272	miR-593	
miR-128	miR-27a	miR-379	miR-200b	miR-132	miR-640	miR-887	
miR-652	miR-93	miR-30a	miR-501-5p	miR-1914*	miR-604	miR-524-5p	
miR-374b	miR-19a	miR-17*	miR-144*	miR-891b	miR-577	miR-1224-3p	
miR-223	miR-484	miR-299-5p	miR-205	miR-1538	miR-602	miR-564	
miR-28-3p	miR-22	miR-148a*	miR-1227	miR-589*	miR-200b*	miR-488*	
miR-377	miR-197	miR-99a	miR-411	miR-650	miR-1909	miR-622	
miR-146a	miR-106b	miR-144	miR-654-3p	miR-770-5p	miR-1269	miR-943	
miR-744	miR-23a	miR-409-5p	miR-194	miR-571	miR-636	miR-875-3p	
miR-584	miR-27b	miR-624*	miR-1181	miR-1205	miR-1539	miR-572	
miR-106a	miR-126*	miR-381	miR-214	miR-149*	miR-639	miR-548b-3p	
miR-30d	miR-423-5p	miR-2110	miR-663	miR-1247	miR-455-3p	miR-483-3p	
miR-143		miR-493	miR-1913	miR-509-3p	miR-124	miR-595	
miR-374a		miR-185*	miR-30c-1*	miR-1207-5p	miR-632	miR-653	
miR-24		miR-193a-5p	miR-99a*	miR-373*	miR-615-5p	miR-605	
miR-130b		miR-502-5p	miR-552	miR-194*	miR-138-2*	miR-105*	
miR-191		miR-551a	miR-671-5p	miR-885-5p	miR-2113	miR-141*	
miR-339-5p		miR-582-5p	miR-1972	miR-661	miR-1203	miR-297	

OS denotes osteosarcoma.

Supplementary Table 6: Clinical information of the osteosarcoma plasma samples used for the discovery and validation cohorts

Characteristics		Discovery	Validation
		n =32	*n* = 29
Age at Diagnosis (Year)			
	Median (range)	13 (5–22)	12.7 (5.6–17.3)
	< 10	7 (22%)	5 (17%)
	≥ 10	25 (78%)	24 (83%)
Gender			
	Male	14 (43%)	15 (52%)
	Female	18 (57%)	14 (48%)
Metastatic at diagnosis			
	Yes	11 (34%)	16 (55%)
	No	21 (66%)	13 (45%)
Primary Site			
	Extremities	27 (84%)	28 (96%)
	Others	1 (3%)	1 (4%)
	Unknown	4 (13%)	0 (0%)
Relapse			
	Yes	16 (50%)	12 (42%)
	No	12 (37.5%)	17 (58%)
	Unknown	4 (12.5%)	0 (0%)

In the discovery cohort, the plasma samples were collected from 32 osteosarcoma patients at initial diagnosis who were enrolled from Texas Children's Hospital (TCH, Houston, TX) and collaborating institutions. In the validation cohort, all the 29 osteosarcoma plasma samples were collected and provided by the Children's Oncology Group. Plasma samples were collected from OS patients who consented to participate under an institutional review board–approved protocol.

Supplementary Table 7: Clinical information of the OS tumor samples in the IMGENEX TMA used for the *in situ* hybridization experiment

Characteristics		
		$n = 60$
Age at Diagnosis (Year)		
	Median (range)	18.5 (5–61)
	< 10	3 (5%)
	≥ 10	57 (95%)
Gender		
	Male	44 (73%)
	Female	16 (27%)
Primary Site		
	Extremities	52 (87%)
	Others	8 (13%)
